# Evolutionary Potential of Parthenogenesis—Bisexual Lineages within Triploid Apomictic Thelytoky in *Cacopsylla ledi* (Flor, 1861) (Hemiptera, Psylloidea) in Fennoscandia

**DOI:** 10.3390/insects13121140

**Published:** 2022-12-11

**Authors:** Seppo Nokkala, Valentina G. Kuznetsova, Peppi Pietarinen, Christina Nokkala

**Affiliations:** 1 Laboratory of Genetics, Department of Biology, University of Turku, FI-20014 Turku, Finland; 2 Department of Karyosystematics, Zoological Institute, Russian Academy of Sciences, Universitetskaya nab. 1, 199034 Saint-Petersburg, Russia

**Keywords:** parthenogenesis, apomixis, triploid, evolution, bisexual, Fennoscandia, reproductive isolation, *Cacopsylla ledi*, peripatric speciation

## Abstract

**Simple Summary:**

The jumping plant louse *Cacopsylla ledi* (Flor, 1861) (Hempitera: Psylloidea) is a triploid apomictic parthenogenetic species. Occasionally, so-called rare males and diploid females that can interbreed with each other are found in the populations. If these bisexuals were able to move to a new area and establish a new population, this could eventually lead to the formation of a new bisexual species through a founder event. By using cytological and molecular approaches, we were able to find a bisexual lineage occurring together with triploid females in northern Fennoscandia and originating from rare males and diploid females, as evidenced by a shared haplotype with triploid parthenogenetic females. An independent bisexual population of parthenogenetic origin was found in the Kola Peninsula. By contrast, another bisexual lineage in southern Fennoscandia carried a different haplotype than the triploids in the same population. This lineage appeared to represent the ancestral bisexual *C. ledi*, which had moved postglacially northwards along with the triploid parthenogenetic females. As these lineages are well separated from each other, the population in the Kola Peninsula has the potential to develop into a new bisexual species refuting the view that parthenogenesis is an evolutionary dead end.

**Abstract:**

A widely accepted hypothesis is that parthenogenesis is an evolutionary dead end since it is selectively advantageous in the short term only but results in lowered diversification rates. Triploid apomictic parthenogenesis might represent an exception, as in favorable environments, triploid females are able to produce rare males and diploid females. The aim of the present study was to analyze the modes of reproduction and their evolutionary implications in the parthenogenetic psyllid *Cacopsylla ledi* (Flor, 1861) from Fennoscandia. The cytogenetic assessment of ploidy levels and the analysis of the *COI* haplotype revealed two geographically separated bisexual lineages implying genuine bisexual populations. The southern lineage occurring south of latitude 65° N in Finland showed a *COI* haplotype different from that of parthenogenetic triploids in the same population but identical to the haplotype of specimens in a genuine bisexual population in the Czech Republic. This allows us to suggest that bisexuals in southern Fennoscandia represent the original bisexual *C. ledi.* By contrast, in the northern bisexual lineage north of latitude 65° N, rare males and diploid females carried the same haplotype as triploids in the same population, having been produced by the triploids. In the Kola Peninsula, a genuine bisexual population of presumably rare male/diploid female origin was discovered. As this population is geographically isolated from populations of the ancestral bisexual *C. ledi*, it can develop into a new bisexual species through peripatric speciation during evolution. Our findings demonstrate that apomictic triploid parthenogenesis is not necessarily an evolutionary dead end but is able to lead to the emergence of a new bisexual species of parthenogenetic origin.

## 1. Introduction

The vast majority of insects reproduce bisexually, and unisexual species that reproduce via parthenogenesis comprise less than 1% of all species [1]. Parthenogenetic species are widely distributed among holometabolous (Holometabola) and non-holometabolous (‘Hemimetabola’) insect taxonomic orders [2,3].

In thelytokous parthenogenesis, females produce daughters from unfertilized eggs without contribution from the males, either through automixis or apomixis. In automictic parthenogenesis, females show normal meiosis and produce haploid gametes; diploidy is restored by the fusion of two haploid gametes, but other mechanisms may also be involved [4,5]. In apomictic parthenogenesis, meiosis is replaced by a modified mitosis, and this type of parthenogenesis means strict clonal reproduction. Apomictic parthenogenesis is usually coupled with polyploidy, most often triploidy, which is thought to be the consequence of hybridization between closely related species [1,6]. It is assumed that the majority of parthenogenetic lineages evolved recently from sexual ancestors [7] and that the transition from bisexual to unisexual taxa arose spontaneously [8].

It is not uncommon that males are encountered at low frequencies, usually below 5%, in populations of parthenogenetic species [9,10,11,12,13]. These occasional, so-called “rare males” are produced by parthenogenetic females from time to time through some unknown mechanisms. According to Palmer and Norton [14] and Norton et al. [15], a population may be considered bisexual if at least 30% of individuals are males. If we accept this reasoning, we can infer the reproductive mode of a given population by determining the frequency of males.

Geographic parthenogenesis is a widely accepted concept, and it has been stated that if there had been a parthenogenetic lineage and a closely related bisexual lineage in one of the refugia during the last glacial maximum, the parthenogenetic lineage would have extended its range towards the north when the ice retreated (reviewed by [16]). This assumption is supported by the fact that parthenogenetic forms are often encountered in marginal areas such as deserts, high altitudes, or high latitudes [16,17]. Very little is known about the evolutionary potential of parthenogenesis. It is commonly suggested that parthenogenetic lineages are short-lived in an evolutionary sense. The evolutionary potential of parthenogenetic species is thought to be low since there are no mechanisms to counteract the effects of accumulating deleterious mutations, which could explain the observed low frequency, less than 1.5%, of thelytokous species among Hexapoda [18]. In this light, it is perhaps somewhat puzzling that parthenogenesis is still so widely distributed throughout the animal kingdom [2,3], as it seems likely that selection should eliminate parthenogenetic lineages efficiently. Moreover, several groups (e.g., bdelloid rotifers and oribatid mites) have been shown to evolve for long periods of time in a strictly parthenogenetic mode, and the most convincing example of this is bdelloid rotifers that have existed for millions of years apparently without sex or meiosis [19,20]. Domes et al. [21] have probably been the only ones to present evidence for the evolutionary potential of parthenogenesis by showing that the bisexually reproducing oribatid mite family Crotoniidae originated from a parthenogenetic ancestor. However, they were unable to explain this unexpected finding.

The best-studied example of the evolutionary potential of automictic parthenogenesis is the diploid brine shrimp *Artemia* [11,22,23,24]. In *Artemia* populations, rare males appear at a low frequency. Males show normal meiosis and produce functional sperm. If these males mate with females of a closely related bisexual *Artemia* species, heterozygotes carrying alleles for parthenogenesis are produced. This can lead to the production of a new parthenogenetic species. Parthenogenesis of this kind is called contagious parthenogenesis [6]. Recent research indicates that contagious parthenogenesis may also rarely be transmitted by parthenogenetic *Artemia* females [24]. The mating of rare males produced by parthenogenetic females with closely allied bisexual females may occur again in succeeding generations, which may be one of the causes explaining the surprisingly high level of genetic diversity observed in various parthenogenetic lineages [22,24,25,26,27].

As far as we know, there is no published documentation on the evolutionary potential of an apomictic parthenogenetic species. The apomictic parthenogenetic jumping plant louse *Cacopsylla myrtilli* (W Wagner, 1947) (Hemiptera, Psylloidea) has been thoroughly studied both at the cytological and the molecular level by Nokkala et al. [12,13,28]. In populations of *C. myrtilli*, triploid apomictic females were shown to produce diploid, non-functional rare males (unable to produce normal haploid sperm) and functional diploid females in equal proportions in every generation as reversals from triploidy. For this reason, diploids share the same *COI* haplotype as triploid parthenogenetic females. The proportion of diploids is dependent on environmental conditions, the highest proportion being produced in high-altitude populations. This property opens a novel view of the evolutionary possibilities in species with apomictic parthenogenesis. If the males produced by parthenogenetic females turned out to be functional and, thus, are able to successfully mate with congeneric diploid “sexual” females, transmitting their genes to the diploid offspring might lead to the emergence of a bisexual population and subsequently to the formation of a new bisexual species [29].

The aim of the present study was to investigate the characteristics of parthenogenesis in another jumping plant louse *Cacopsylla ledi* (Flor, 1861), exhibiting triploid apomictic parthenogenesis and producing functional males in populations, i.e., males showing normal meiosis and producing haploid gametes [29]. *C. ledi* is widely distributed in the northern hemisphere. According to Ossiannilsson [30], the species inhabits a limited area in southeast Norway and is sporadically found in northern Norway, while it is widely distributed in Sweden and western Fennoscandia, the Baltic countries, Poland, Germany, Russia, Japan, and Alaska reflecting the distribution of its host plant. It lives on the shoots and leaves of Labrador Tea *Ledum palustre* L. 1953 (syn. *Rhododendron tomentosum* Harmaja, 1990) and is univoltine, i.e., has a single generation per year. In contrast to *C. myrtilli*, in a *C. ledi* population from Turku, the *COI* haplotype of infrequent males and diploid females was different from that of parthenogenetic triploid females, showing that these diploids could not have been produced by triploid females [29]. The purpose of the present study focused on *C. ledi* with the aim of (1) finding out the origin of diploids carrying different haplotypes from that which is present in triploid parthenogenetic females within a population, and (2) study populations from Finland, Northwest Russia, Norway, and Sweden to find out if rare males sharing the same haplotype with triploid females could be found. We combined cytogenetic methods for distinguishing between triploid and diploid females and molecular analysis for determining the *COI* haplotype.

## 2. Materials and Methods

### 2.1. Samples

**Figure 1:**
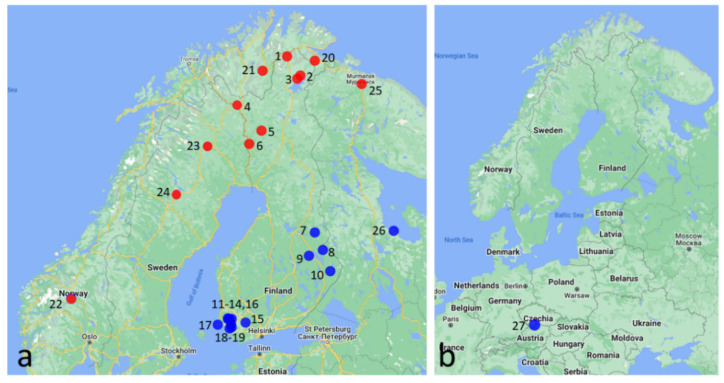
Sampling sites of *C. ledi* in (**a**) Fennoscandia and (**b**) Czech Republic. Numbers correspond to the numbers of locations presented in Table 1. Red dots: genuine parthenogenetic populations and bisexual populations carrying haplotype H1. Blue dots: populations where diploid females and males carrying haplotype H2 reproduce bisexually.

Samples of *Cacopsylla* specimens were collected in 4130 locations in Finland, four in Norway, four in Sweden, two in Russia, and one in the Czech Republic from the food plant *L. palustre* during 2017–2022 and were stored in 96% alcohol (Table 1, Supplementary Table S1). When checked in the laboratory, samples were found to include either *C*. *ledi* only (20 samples) or *C. borealis* Nokkala and Nokkala, 2019 only (1 samples) or both (6 samples). Data from Nokkala et al. [29], which triggered the present study, were also included. Locations of the *C. ledi* samples analyzed in this study are presented in Figure 1.

### 2.2. Cytological Study

Before performing the haplotype study, we determined the ploidy level of the females. For this, we analyzed metaphase I (MI), which, as we showed previously [29], can be found in mature eggs taken from females collected in mid-August or later. Similarly, males were first analyzed cytologically to find out if meiosis was normal or aberrant [12,13,29]. Male meiosis can be studied throughout the season as long as the males are available. Alcohol-preserved females and males were dissected, and the abdomen was immersed overnight in a 3:1 (ethanol: acetic acid) fixative for cytological analysis, while the head and thorax were preserved in alcohol for subsequent molecular analysis of the same individual. After overnight fixation, the abdomen was transferred to a drop of 45% acetic acid on a microscopic slide, and 4–5 mature eggs were extracted from the females and cut into halves using sharpened tungsten needles. When the yolk became transparent, an 18 × 18 mm coverslip was laid on top of the material. After squashing, the slide was left at room temperature for 4–5 min before transferring onto dry ice. Following the removal of the coverslip, the slides were dehydrated in fresh 3:1 fixative for 30 min and air-dried. The slides were stored in a dust-free place and subsequently stained first in Schiff’s reagent for 20–25 min after 6 min of the hydrolysis stage, followed by 5% Giemsa staining for 30 min. For analyzing the male meiosis, testes were dissected and cut into pieces in 45% acetic acid, and the procedure was continued as with the female slides, except that the staining with Schiff´s reagent was for 10–15 min and staining with 3% Giemsa was for 20 min.

### 2.3. Molecular Analysis

The total genomic DNA was extracted using the DNeasy Blood & Tissue Kit (Qiagen, Hilden, Germany), as described in our previous studies [13,29,31]. After homogenization in an extraction buffer, the procedure was completed following the manufacturer’s instructions. PCR amplification of a fragment of the *cytochrome c oxidase subunit I* (*COI*) gene was carried out using the primer pair HybCacoCO (5′-T7Promoter(F)-CTAACCATAARACTATTGGAAC-3′) and HybHCOMod (5′-T3Promoter(R)-TAAACTTCAGGGTGACAAAAAATCA-3′) as described earlier [29,31]. PCR products purified with QIAquick PCR Purification Kit (Qiagen, Hilden, Germany) were sent to Macrogen Europe (Amsterdam, the Netherlands) for Sanger sequencing. Sequences were edited and aligned with BioEdit 7.2.0 software [32] and checked by visual inspection. Processed haplotypes were compared with haplotype Turku 1, accession number MF978762, where nucleotide #191 is T, and haplotype Turku 2, accession number MF978763, has a nucleotide #191 of A. In the present study, haplotype Turku 1 (191T) is referred to as H1, and haplotype Turku 2 (191A) as H2.

## 3. Results

Altogether 4051 specimens of *C. ledi* were collected from 27 populations. Most specimens (~85%) were females; male frequency among the different populations varied between 0–46.0% (Table 1).

In the first stage of the research, we aimed to study how widespread the populations were in south and southwest Finland, where males showed a haplotype different from that of triploid females (populations 11–19 in Table 1 and Figure 1). The frequency of males in those populations varied from 2.2% to 46.0%.

**Figure 2:**
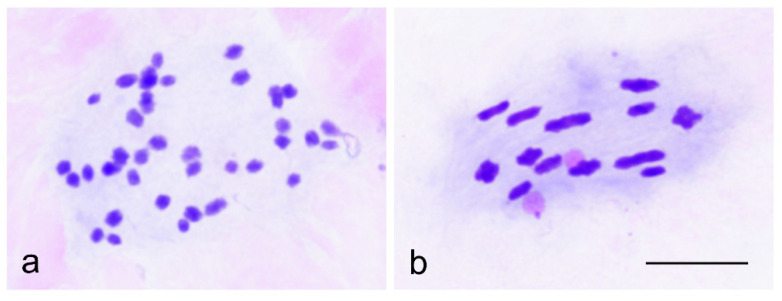
(**a**,**b**). Prometaphase in mature eggs of female *C. ledi*. (**a**) Triploid apomictic female showing 39 univalents. (**b**) Diploid female, prometaphase I, showing 13 bivalents. Bar equals 10 µm.

Three of these populations were studied cytologically (Table 2), including one bisexual population where the male frequency was 37.0% (Pöytyä, Lammenrahka), one population where the male frequency was 13.3% (Eura Laustinrahka), and one population with a low frequency of 4.7% of males only (Pöytyä, Levonsuo). The cytological study of oogenesis allowed differences between triploid and diploid females to be distinguished (Figure 2). Triploid females showed 39 univalent chromosomes (36A + XXX) (Figure 2a), and diploid females showed 13 bivalents (12AA + XX) (Figure 2b). In diploid females, chiasma formation was random, as indicated by their distal to a medial location in different bivalents. The frequency of diploid females and males was similar at low, medium, and high levels of male frequency. In Pöytyä, Lammenrahka population, the haplotype of all males was the same as in the diploid females (H2). In Eura, Laustinrahka and Pöytyä, Levonsuo populations, two and one males, respectively, shared the same haplotype with triploid females (H1), thus representing genuine “rare males” produced by triploid females, whereas most males shared the same haplotype with diploid females (H2) (Table 2 and Table 3). It may be inferred that males and diploid females with the H2 haplotype represent a bisexually reproducing lineage, while triploid females with the H1 haplotype reproduce via parthenogenesis. Both modes of reproduction may occur within a population. Cytological analysis revealed that if the haplotype of triploid and diploid females was different, diploid females could be recognized by haplotype analysis alone, which makes chromosomal analysis unnecessary.

**Figure 3:**
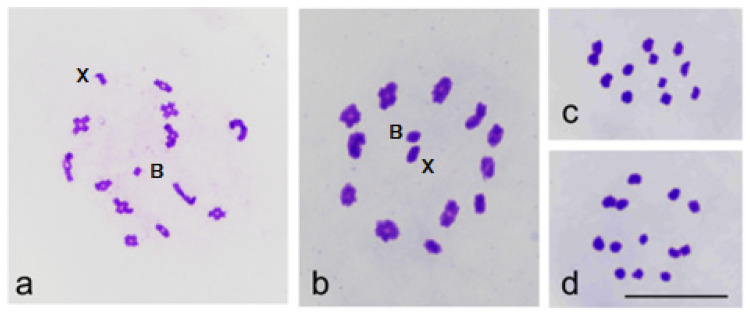
(**a**–**c**) Male meiosis in *C. ledi*. (**a**) Diplotene, 12 bivalents and univalent X and B chromosomes. (**b**) Metaphase I, X and B chromosomes in the center of the radial metaphase plate. (**c**) Metaphase II, n = 12. (**d**) Metaphase II, n = 12 + X.

In male meiosis, prophase I showed 12 bivalents, the X chromosome, and commonly one B chromosome (Figure 3a). Metaphase I showed 12 bivalents and a univalent X chromosome, or if a B chromosome was present, it was located along with the X in the center of a radial metaphase plate (Figure 3b). At metaphase II, either 12 (Figure 3c) or 12 + X chromosomes were observed when there was no B chromosome. (Figure 3d).

In the second stage of the research, we examined populations from eastern Finland and northwest Russia (populations 2–10 and 25–26 in Table 1 and Figure 1) to study the distribution of the bisexually reproducing lineages on a wider geographical scale (Table 3). Both males and females (haplotype H2) were found in the Kuhmo and Nurmes populations in Finland. The Kuhmo population (pop. 7 in Table 1) with a high male frequency (28.0%) was analyzed in more detail. All 35 males examined carried the H2 haplotype. Of the 41 females examined, 17 females carried the H2 haplotype, whereas the remaining 24 females carried haplotype H1. Haplotype H2 was also represented by one female out of a total of 18 examined in the Russian Kolezma population (pop. 26 in Table 1) located at the White Sea region relatively close to Kuhmo. This suggests that bisexuals with haplotype H2 could be of a refugial origin. To test this hypothesis, we analysed a bisexual population (pop. 27 in Table 1) from the Czech Republic. In this population, both females and males were found to carry the H2 haplotype. Thus, it seems evident that diploids carrying the H2 haplotype represent the ancestral bisexual form of *C. ledi*.

In Finland, populations have also been found where males and diploid females show the same haplotype H1 as triploid females (pop. 2, 3, and 5 in Table 1 and Figure 1). Cytological analysis of the Sevettijärvi population revealed 36 triploid females, 5 diploid females and 7 males (Table 2), all showing the H1 haplotype. These diploids represent genuine “rare males” and diploid females, and their frequencies show no statistical difference from each other (χ^2^ = 0.2858, N.S.). One sample from northern Finland (Kittilä) and one sample from northern Norway (Neiden, Jäälä) were all-female parthenogenetic populations.

To our surprise, in a sample from the Kola Peninsula, Mokhnaktina Pakhta (pop. 25 in Table 1), the male frequency was 36.5%, suggesting that the population was bisexual, but the male haplotype was H1, i.e., the same as in triploid females. Additionally, in Norway, in the Mohkejogas population (pop. 21 in Table 1), where the male frequency was 12.8% (comparable to that in Eura, Laustinrahka population), it is plausible that at least part of diploids reproduced bisexually, the frequency of males being too high for “rare males”. This ”northern” bisexual lineage with haplotype H1 is clearly different from the refugial-origin bisexual lineage with haplotype H2. These lineages show exclusive geographical distributions where all diploids south of latitude 65° N carry haplotype H2, while diploids north of latitude 65° N carry haplotype H1. It is worth remembering that in most populations (12/17 locations in Finland, 17/25 total) sampled north of latitude 65° N, predominantly, only specimens of *C. borealis* were encountered.

## 4. Discussion

We have established that *Cacopsylla ledi* displays an extremely complex population structure in Fennoscandia. Aside from all-female populations consisting of triploid parthenogenetic females, two distinct diploid lineages were found, which usually formed mixed populations with triploids or, in some locations, existed as diploid bisexual populations. Besides *C. ledi*, the related species *C. borealis* utilise *Ledum palustre* as a food plant and thus competes for the same habitats, especially in northern Fennoscandia, where in most locations, samples were found to consist predominantly or exclusively of *C. borealis* specimens (Table S1). However, the distribution of *C. borealis* does not reach areas south of latitude 63° N [31].

### 4.1. Diploid Males and Females Show a Haplotype Different from Triploid Females

We have shown that in southern/southwestern populations in Finland, diploids carry a different haplotype (H2) from triploid females (H1) and are present in populations with varying frequencies [29]. In two populations, Pöytyä, Lammenrahka, and Pöytyä, Valastensuo, the male frequency was 37% and 46%, respectively, which is an unmistakable indication of the bisexual reproduction [14,15] of diploids occurring in these populations. This does not totally exclude some amount of parthenogenetic reproduction in the same populations. Actually, in the Lammenrahka population, one female out of 45 analyzed was found to be triploid. It is obvious that even if the frequency of diploids is low, they do reproduce bisexually within populations consisting mostly of parthenogenetic triploids. This bisexual lineage carrying haplotype H2 is also present in eastern Finland south of latitude 65° N in Kuhmo and in Russia in the White Sea region of Kolezma. In fact, diploids with the haplotype H2 have not been found north of latitude 65° N at all. Thus, it can be inferred that these diploids represent a bisexual lineage that has distributed into Finland from the east during the post-glacial recolonization process. To confirm this, we also sampled a genuine bisexual population of *C. ledi* from the Czech Republic in a location known as a northern cryptic refugium [33,34,35,36] which revealed that all specimens carried haplotype H2. This is strong evidence that the diploid lineage characterized by haplotype H2 represents the ancestral bisexual *C. ledi,* which has participated in the formation of the triploid parthenogenetic lineage of *C. ledi*. The bisexual *C. ledi* recolonized northern latitudes alongside the parthenogenetic *C. ledi*.

### 4.2. Diploids Show the Same Haplotype as Triploid Parthenogenetic Females

Although triploids, both in southern and northern Finland, carry the same haplotype H1, triploids in the south (south of latitude 65° N) produce rare males (H1) only occasionally and at a low frequency. However, in the north (north of latitude 65° N), triploids were found to produce males and diploid females carrying haplotype H1 at a higher frequency, as in the Sevettijärvi population. In the Mohkejogas population (Finnmark, Norway), the male frequency was 12.8%, i.e., much higher than the frequency of “rare males” in general, indicating that some bisexual reproduction is present within this population. Most importantly, in Mokhnaktina Pakhta (Murmansk), a bisexual population (male frequency 36.5%) was found. The diploids in this population showed haplotype H1 and had originated from rare males and diploid females produced by triploid females.

### 4.3. How Are the Bisexually Reproducing Populations Formed?

It is rather common that apomictic triploid *Cacopsylla* species may produce rare males and diploid females as reversals from triploidy; this property depends greatly on environmental factors [13]. For instance, in *C. myrtilli*, the highest number of rare diploids, both males and females (10% of each), was found at the altitude of 1100 m a.s.l. in an alpine habitat in southern Norway [13]. To explain the existence of bisexual populations with very high frequencies of males (around 40%), factors other than the environmental need to be considered. The postglacial recolonization process is tightly coupled with leading-edge expansion (see [37]), from which the colonization process was started by long-distance dispersers that set up colonies and quickly expanded to suitable habitats before others arrived. This kind of colonizing process would occur repeatedly along the colonization route and, accompanied by founding events, would subsequently lead to a loss in genetic diversity [38]. The existence of populations of *C. ledi* with a high frequency of diploid females and males can be explained by the repeated setting up of new colonies where, by chance, at some stage(s) of expansion, the founders of a new colony would be comprised mostly (or entirely) of diploids. This leading-edge expansion model accounts well for the formation of bisexually reproducing populations of both haplotype H1 origin in the Kola Peninsula and haplotype H2 origin in southern Finland.

### 4.4. Evolutionary Potential of Triploid Apomictic Parthenogenesis in C. ledi

A bisexually reproducing population, “Mokhnatkina Pakhta”, in the Kola Peninsula near Severomorsk at latitude 69° N, is of parthenogenetic origin. This population is most crucial in terms of the evolutionary potential of a parthenogenetic species. The population is geographically well isolated from the ancestral diploid *C. ledi*, the nearest occurrence of which is at the latitude 65° N in Kuhmo, Finland, and in Kolezma, Russia. As gene flow between these two diploid lineages was completely prevented, the “Mokhnatkina Pakhta” population has an excellent possibility to develop into a new diploid species of parthenogenetic origin during the course of evolution via peripatric speciation, which is a special subtype of allopatric speciation.

A crucial question is how and when this geographic isolation was formed. It seems apparent that it is somehow connected to the post-glacial recolonization process. It seems likely that the ancestral bisexual *C. ledi* species arrived north substantially later than both parthenogenetic *C. ledi* and *C. borealis*. The expansion of the parthenogenetic *C. ledi* resembled that of *C. myrtilli* [39], although reaching further south, whereas *C. borealis* expanded towards the north. The expansion of bisexual *C. ledi* species reached the White Sea region only when the most suitable habitats in northern Fennoscandia were already occupied by the parthenogenetic *C. ledi* and especially the efficient parthenogenetic colonizer C. *borealis*. The newcomer is distributed towards the south and southwest Finnish peninsula, the last areas to become available after the last glacial maximum [37,40,41,42,43] and where *C. borealis* was absent and not competing with it for habitats. This phenomenon is well recognized in the leading-edge expansion model, i.e., when earlier settlers had occupied suitable habitats, it was much more difficult for the newcomers to move forward [41,44]. Our findings evidence that the sexual traits (reviewed by [45]) in *C. ledi* are successfully maintained under apomictic triploid parthenogenesis in both males and diploid females. However, this property seems to be quite species-specific, as in the closely related *C. mytilli,* whose sexual traits decayed under parthenogenesis in males as they were non-functional [12].

## Figures and Tables

**Table 1:** Sampling locations, number of specimens, male frequency, and collection date of *Cacopsylla ledi* analyzed in the present study.

Location		Coordinates	Females	Males	Male%	Date
**Finland**						
1	Utsjoki, Hietala	69.851667	28	2	6.7	15 August 2017
		27.009444				
2	Inari, Nitsijärvi	69.302	41	3	7.3	14 August 2021
		28.107				
3	Inari, Sevettijärvi	69.2161111	160	10	5.9	17 August2017
		27.8705556				
4	Enontekiö, Käsivarrentie	68.32450	11	0	0	14 August 2022
		22.993397				
5	Kittilä	67. 628	184	0	0	12 August 2021
		24.933				
6	Kolari	67.209167	51	1	1.9	9 August 2018
		23.906389				
7	Kuhmo, Syväjärvi	64.1946	161	64	28.4	4 August 2019
		29.2932				
8	Lieksa, Nurmijärvi	63.5430	77	1	1.3	5 August 2019
		29.9635				
9	Nurmes, Panjavaara	63.335394	94	6	6.0	1 August 2020
		28.828826				
10	Ilomantsi, Tokrajärvi	62.7544	68	0	0	5 August 2019
		30.5773				
11	Eura, Isosuo	60.901389	107	18	14.4	24 August 2018
		22.161944				
12	Eura, Laustinrahka	60.889722	85	13	13.3	24 August 2018
		22.146667				
13	Pöytyä, Levonsuo	60.886111	102	5	4.7	15 August 2019
		22.444722				
14	Pöytyä, Valastensuo	60.856000	34	29	46.0	21 August 2020
		22.273000				
15	Tammela, Torronsuo	60.738889	56	6	9.7	18 July 2019
		23.585778				
16	Pöytyä, Lammenrahka	60.676944	182	107	37.0	22 August 2018
		22.425556				
17	Kustavi, Kaurissalo	60.655556	313	7	2.2	25 August 2019
		21.303333				
18	Marttila, Onnenperänrahka	60.544444	202	27	11.8	19 August 2019
		22.444722				
19	Turku, Runosmäki	60.498889	1141	100	8.8	[29]
		22.265278				
**Norway**						
20	Neiden, Jäälä	69.732586	42	0	0	28 July 2020
		29.296901				
21	Mohkejogas, Finmark	69.443289	218	28	12.8	26 July 2020
		25.193346				
22	Sjoa, Stålane	61.6875000	3	0	0	1 August 2009
		9.2408333				
**Sweden**						
23	Jokkmokk, Kåbdalis	66.027386	9	0	0	14 August 2022
		19.908879				
24	Sorsele, Europaväg 45	65.571807	12	0	0	14 August 2022
		18.045919				
**Russsia**						
25	Murmansk, Mokhnatkina Pakhta	69.050851	115	66	36.5	31 July2020
		33.161461				
26	Kolezma	64.246033	44	0		29 September 2020
		35.813505				
**Czech Republic**						
27	Cervene Blato Natl. Nat. Res.	48.858611	10	8	44.4	20 July 2021
		14.803889				

**Table 2:** Distribution of haplotype H1 (191T) and haplotype H2 (191A) in cytologically studied *Cacopsylla ledi* individuals.

Population (Freq. of Males)		3n Females	2n Females	2n Males	Principal Type of Reproduction
1	Inari, Sevettijärvi (5.9%)	H1 N = 36	H1 N = 5	H1 N = 7	Parthenogenetic
2	Pöytyä, Lammenrahka (37.0%)	H1 N = 1	H2 N = 43	H2 N =30	Bisexual
3	Eura, Laustinrahka (13.3%)	H1 N = 16	H2 N = 23	H1 N = 2 H2 N = 11	Mixed
4	Pöytyä, Levonsuo (4.7%)	H1 N = 44	H2 N = 1	H1 N = 1 H2 N = 4	Mixed

**Table 3:** The number of individuals carrying haplotypes H1 and H2, and type of reproduction in *Cacopsylla ledi* samples sequenced.

Sampling Location	Females			Males		Type of Reprouction
	3n	2n				
	H1	H1	H2	H1	H2	
Inari, Nitsijärvi				2		parthenogenetic
Inari, Sevettijärvi	36	5		7		parthenogenetic
Enontekiö, Käsivarrentie	11					parthenogenetic
Kittilä	10					parthenogenetic
Kolari	4			1		parthenogenetic
Kuhmo, Syväjärvi	24		17		35	mixed
Nurmes, Panjavaara					6	mixed
Eura, Isosuo					7	mixed
Eura, Laustinrahka	16		23	2	11	mixed
Pöytyä, Levonsuo	44		1	1	4	mixed
Pöytyä, Valastensuo			11		11	bisexual
Tammela, Torronsuo					6	mixed
Pöytyä, Lammenrahka	1		43		30	bisexual
Kustavi, Kaurissalo	6				6	mixed
Marttila, Onnenperänrahka					16	mixed
Turku, Runosmäki	61		6		5	mixed
Jokkmokk, Kåbdalis	9					parthenogenetic
Sorsele, Europavägen 45	12					parthenogenetic
Neiden, Jäälä	11					parthenogenetic
Mohkejogas, Finmark	20			12		mixed
Sjoa, Stålane	2					parthenogenetic
Murmansk, Mokhnatkina Pakhta		30		30		bisexual
Kolezma	17		1			mixed
Cervene blato Natl.Nat.Res.			6		2	bisexual

## Data Availability

Haplotype sequences referred to in this study are deposited in GenBank under the accession numbers MF978762- MF978763.

## Supplementary Materials

The following supporting information can be downloaded at: https://www.mdpi.com/article/10.3390/insects13121140/s1, Table S1: List of samples with *C. borealis*.
